# Control of triboelectric charges on common polymers by photoexcitation of organic dyes

**DOI:** 10.1038/s41467-018-08037-5

**Published:** 2019-01-17

**Authors:** S. Doruk Cezan, Atakan A. Nalbant, Muhammed Buyuktemiz, Yavuz Dede, H. Tarik Baytekin, Bilge Baytekin

**Affiliations:** 1 Department of Chemistry, Bilkent University, 06800 Ankara, Turkey; 2 Department of Chemistry, Faculty of Science, Gazi University, 06500 Teknikokullar, Ankara, Turkey; 3 UNAM-National Nanotechnology Research Center, Bilkent University, 06800 Ankara, Turkey

## Abstract

Triboelectric charging of insulators, also known as contact charging in which electrical charges develop on surfaces upon contact, is a significant problem that is especially critical for various industries such as polymers, pharmaceuticals, electronics, and space. Several methods of tribocharge mitigation exist in practice; however, none can reach the practicality of using light in the process. Here we show a light-controlled manipulation of triboelectric charges on common polymers, in which the tribocharges are mitigated upon illumination with appropriate wavelengths of light in presence of a mediator organic dye. Our method provides spatial and temporal control of mitigation of static charges on common polymer surfaces by a mechanism that involves photoexcitation of organic dyes, which also allows additional control using wavelength. This control over charge mitigation provides a way to manipulate macroscopic objects by tribocharging followed by light-controlled discharging.

## Abstract (web summary)

Contact charging of insulators is a significant problem for various industries, such as plastics, electronics, and space. Here the authors gain spatial and temporal control of discharge of triboelectrically charged polymers upon illumination of a set of common organic dyes.

## Introduction

Electrical charges are formed and retained on insulator surfaces through contact and friction. This interesting phenomenon, called as contact electrification or tribocharging^1–6^, has many corollaries, the most important one being the building up of a large electric potential on surfaces. Technological endeavors promoting this electrical potential has given rise to interesting applications such as electrostatic separations^7^ and triboelectric generators^8^. However, the generation of this surface electrical potential also causes stiction or electrostatic discharges on insulator surfaces, which are significant problems that affect many billion-dollar-industries such as plastics, pharmaceuticals, electronics, and space^9^. Therefore, innumerous studies have been dedicated to permanent prevention of tribocharges. Such permanent prevention can be achieved by addition of conductive materials (e.g., carbon, metals, or conductive polymers)^9^, rendering surfaces hydrophilic and hence more conductive (due to water layer on surfaces)^10^, synthesizing special antistatic (co)polymers^11^, or doping the polymers with radical-scavenging molecules for destabilizing the charges^12,13^.

The controlled mitigation of charges (rather than the permanent mitigation described above) is mainly used in electrophotography and laser printing^14^, and is currently only possible through special materials such as photoconductive polymers, which limits the development of other possible applications. Electrophotography uses light, which is the most straightforward stimulus for controlling biological, chemical, and physical phenomena, to control (dis)charging. Here we display a method to mitigate the tribocharges using light stimulus, as in electrophotography, but this time the method is not restricted to photoconductive polymers and is applicable to a broad set of common polymers.

In addition to possible technological applications, the control of electrical charges, i.e., turning them off when desired rather than permanently removing them, may also provide a way to obtain useful work, such as for manipulation of small objects or for charge patterning. Current approaches to charge mitigation, such as to increase conductivity of surfaces by metal or ion doping fail to provide the above-described controllability, unless the material being charged is specially synthesized to have switchable characteristics upon illumination^15,16^. For conventional polymers, a more general method is required to adapt such a control. We recently developed a charge mitigation methodology^12^ based on the latest findings on the mechanism of charge formation, which suggests that, upon contact, mechanically initiated bond-breakages produce ions^17^ (the tribocharges responsible for the accumulated electrical potential) and radicals^18–26^, which also play a major role in electrification^12,26^. In this new method, charge mitigation is possible through chemical removal of the formed radicals. Although this method is exceptional in preserving the electrical and mechanical properties of the material, it, too, provides only a permanent antistatic property to the material. In this study, to create a “switch”, we use organic dyes that are “placed” in vicinity of the mechanochemically formed species (i.e., radicals, anions, and cations). This provides a temporal (when the discharging is desired) and spatial (on desired loci on polymer samples) control of tribocharging on polymer surfaces, in addition to control with the wavelength of the light stimulus as described below.

## Results

### Light controlled discharge of tribocharged common polymers

**Fig. 1:**
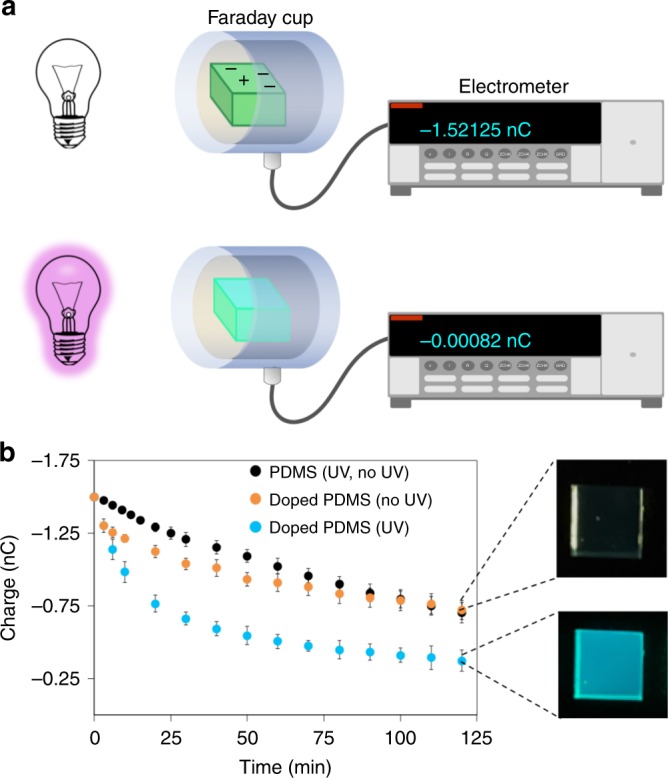
Light controlled discharging of tribocharged polymers **a** Coumarin 6 (C6)-doped (1 × 10^−5^ M) and undoped poly(dimethylsiloxane) (PDMS) pieces (1 cm × 1 cm surface area) are contact-charged by touching to an aluminum foil (Supplementary Figure 1) and tribocharges on the pieces are let to decay in a homemade Faraday cup connected to an electrometer. **b** Unless the pieces in the Faraday cup are illuminated with ultraviolet (UV) light, doping with a dye does not alter decay profiles significantly; C6 doped PDMS pieces (orange dots) and undoped pieces (black dots) have similar rate constants, 0.87 × 10^−4^ s^−1^ and 1.04 × 10^−4^ s^−1^, respectively (Supplementary Figure 2). Illumination of the undoped pieces does not result in any change in the decay rate, either (data overlaps with black dots). However, upon illumination with UV light, tribocharges decay significantly faster on the C6-doped PDMS pieces (blue dots) than all other cases. Error bars correspond to standard deviations determined from at least four independent experiments for every condition. See Methods for further experimental details on sample preparation and Supplementary Figure 2 for data acquired from charge decay experiments. Source data are provided as a Source Data file

The starting point of our study was doping poly(dimethylsiloxane) (PDMS) pieces with organic fluorescent dyes. We hypothesized that the dye may act as a mediator to interact with the mechanospecies (ions or radicals) produced during the mechanical action (contact or rubbing) of the polymer surfaces upon tribocharging. We chose 3-(2-Benzothiazolyl)-7-(diethylamino)coumarin (coumarin 6, C6), a fluorescent dye with a broad ultraviolet (UV) absorption band and absorbance maximum at 427 nm, to dope some of our PDMS pieces (1 cm × 1 cm × 0.5 cm, PDMS, Sylgard 184, Dow Corning, for details on preparation and doping of the pieces, see Methods), which can be molded in polystyrene dishes to provide a decent flat surface for an analysis of typical tribocharging and tribocharge decay on polymer surfaces^12,27^. We also kept some of the pieces undoped for control experiments. All polymer pieces, including controls, were then contact-charged by touching clean aluminum foil surfaces several times. The dye-doped pieces acquired less charge (about 20% less in the max. doping concentrations) than the undoped pieces presumably because of the increase in polarity of the medium by dye addition, since the increase in the dye concentration intensified this effect; Supplementary Fig. 1). The charge decay curves were then recorded by immersing the individual pieces in a homemade Faraday cup connected to an electrometer (Fig. 1a), which showed that the decay rate of tribocharges on the PDMS pieces without the dye (controls) were the same in presence or absence of the light (1.04 × 10^−4^ s^−1^) (Fig. 1b). There was only a slight change in the decay rates of the pieces that were doped with the dye, in ambient light (0.804 × 10^-4^ s^−1^), in comparison with those of the undoped controls. However, tribocharges decay very rapidly, when the dye-doped pieces were illuminated by a UV lamp providing an emission with wavelength matching to the absorption band of the C6 dye. This rapid decay showed a deviation from the expected first order kinetics of tribocharge decay on PDMS surfaces^27^ and implies a presence of (at least) a second party in the decay process (Supplementary Figure 2). Therefore, we surmised that the added dyes act as a mediator in the discharging process.

**Fig. 2:**
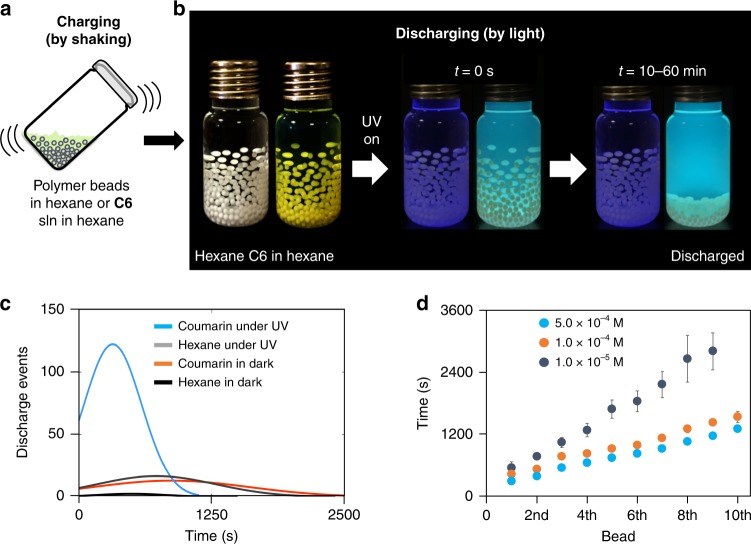
Light controlled discharging of tribocharged polymer beads. **a** Poly(tetrafluoroethylene) (PTFE) beads in hexane or in a coumarin 6 (C6) solution (5 × 10^−4^ M, dry hexane) are tribocharged by shaking on a vortexer for 1 min. (In the photo, 120 beads/vial were used for better visualization of the event, in a typical experiment only 40 beads/vial were used). **b** The charged beads “stick” electrostatically to the walls of the vials and stay stuck on the walls for hours to days. When the vials are illuminated with a UV lamp, only the beads in C6 solution discharge (within a few minutes) and fall to the bottom of the vial. The charge/discharge events can be repeated for hundreds of cycles without any significant change in the results, the data compiled after six individual runs with 40 beads each is shown in **c**. Data from five independent experiments was collected (a total of 200 beads for each experiment, from which only the beads displaying a “discharge event” were counted); curve fit on data collected in experiments was made by Matlab 2017 program using distribution function. For experimental details see Methods and Supplementary Movies 1 and 2. **d** Discharging times of first 10 beads in the experiments in **b**. Error bars correspond to standard deviations determined from six independent experiments. Source data are provided as a Source Data file

Although in the above example, the doping of solid polymer pieces with a fluorescent dye (which can be extended to many other polymers as we have shown before for other organic molecules^12^) shows a successful light-induced discharge, it does not provide a practical medium for studying the parameters (charging medium, different dyes, polymer type etc.) that affect the discharging. To uncover the role of dye in the discharging process, we then used another setup, in which we can probe these important parameters more practically. In a typical setup for visualization of the light-initiated discharging of tribocharged polymers, we introduced 40–120 polymer beads (polytetrafluoroethylene (PTFE), 1.6 mm) in a 20 mL glass vial together with 15 mL dry hexane. (We used 40 beads in each vial in the later experiments for statistics. See Methods for further details on the setup and also Supplementary Figure 3 for results on other vial materials.) The polymer beads in hexane were shaken by a vortexer for 0.5–2 min, and as a result, they were contact-charged (Fig. 2a, Supplementary Movie 1). The net charges on the beads were measured to be −170 ± 55 pC (See Supplementary Fig. 4 on the details about this charge measurement and the proof of the insignificance of solvent charging that might take place during the vigorous agitation.) Charged beads stuck to the inner walls of the glass vial because of electrostatic adhesion. (Fig. 2b; Supplementary Movies 1 and 2, for experimental details, see Methods). In such a setup, once the beads are charged, their charge is preserved for hours to days in absence of external mechanical agitation. (Tribocharged PTFE surfaces retain their triboelectric charge for a year if they are electrically well isolated!). Hexane, with dielectric constant of 1.89 (at 20 °C) provides a medium for efficient charging of the beads and also dissolves the dyes used in the experiments (see below). We have also shown that other solvents with low dielectric constants can also be used in the experiment to see a similar effect; although the beads’ discharge times in these solvents may differ (Supplementary Figure 5). To enhance this sticking and prevent fast discharging due to presence of small amounts of water, dry hexane was used in this initial experiment. (In all further experiments conventional high performance liquid chromatography (HPLC) grade hexane from Sigma Aldrich was used without drying, all vials in the comparison experiments were simultaneously prepared, and the relative humidity (RH) value of the preparation media was kept at 25–35%.). The shaking (charging) time can be optimized to get maximum amount of charge on the polymer beads, we found 1 min to be enough to get most beads adhere to the walls of the glass vial. In another vial, a 5 × 10^−4^ M hexane solution of Coumarin 6 (C6), was introduced together with the polymer beads. Shaking the beads in the solution with the vortexer charged the beads in a similar manner that was observed for the case with pure hexane, i.e., beads remained on the vial walls for hours. However, when the C6 solution was illuminated by a UV lamp, beads fell to the bottom of the vial rapidly, implying that the electrostatic adhesion was ceased because of the mitigation of tribocharges on the beads (Fig. 2b, c, Supplementary Movie 2, final net charge on the beads were measured as −68 ± 2 pC). The observed discharging times (under illumination) was found to decrease with increasing light intensity (Supplementary Figure 6), and increase slightly as the number of beads used in the experiments are increased (Supplementary Figure 7).

Next, in order to confirm that the discharging is related to the presence of the dye, we decreased the dye concentration of the solutions, in which the beads are shaken, from 5 × 10^−4^ M to 1 × 10^−4^ M, and then to 1 × 10^−5^ M, and monitored the time passed for each bead to lose significant amount of its charge and fall down to the bottom of the vial (discharging time). The discharging times were found to be inversely proportional to dye concentration (Fig. 2d), proving the vital presence of the dye for the discharging mechanism—a similar behavior we saw in the case for solid PDMS pieces in the very first experiments (Fig. 1).

**Fig. 3:**
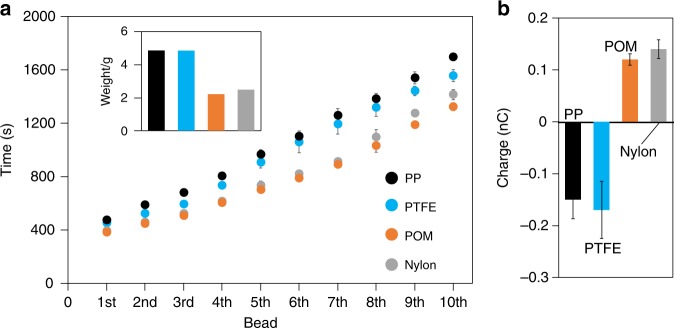
Universality of light-induced discharging for common polymers. **a** Polymer beads made up from different polymers, poly(tetrafluoroethylene) (PTFE), nylon, poly(oxymethylene) (POM), polypropylene (PP) all discharge in Coumarin 6 (C6) solutions upon illumination with 366 nm UV light (Bead diameter = 1.6 mm, 1 min charging time in 5 × 10^−4^ M **C6**). The differences in the discharge times emerge from the different weights (a, inset) and **b** initial charges of the polymer beads. Source data are provided as a Source Data file

The dye-mediated light induced discharging of tribocharged PTFE beads (displayed in Fig. 2) was found to be applicable to many common polymers (Fig. 3): polypropylene (PP), poly(hexamethylene adipamide) (Nylon), or polyoxymethylene (POM) beads in hexane solutions of C6 were tribocharged as described above (all polymer beads were 1.6 mm diameter, Engineering Laboratories Inc., average net charge on beads in air were −150 ± 37 pC, 140 ± 18 pC, 120 ± 12 pC, respectively). Then the electrified beads in the glass vials of the **C6** (hexane) solutions were illuminated with UV light. In each experiment, upon illumination, beads discharged in a similar manner to each other, regardless of the initial net charge on them, Fig. 3a. We believe, this similarity stems from the fact that all polymers charge alike at the molecular/nano scale with both positive and negative charge domains^18,28^—the net charge on the polymers (a few nC cm^−2^) reflects only a small excess of either of these positive or negative charge domains^18^. Small differences between the discharging times were presumably a result of an interplay between the average net charge on the different type of beads and their weights, Fig. 3b. Also, when the glass walls of the shaking medium were covered with polyoxymethylene (POM) and polyethyleneterephthalate (PET) sheets, the photo-initiated discharging still takes place, with some minor differences in the discharging times of the tribocharged beads (Supplementary Figure 3).

### Wavelength control in light induced discharging

**Fig. 4:**
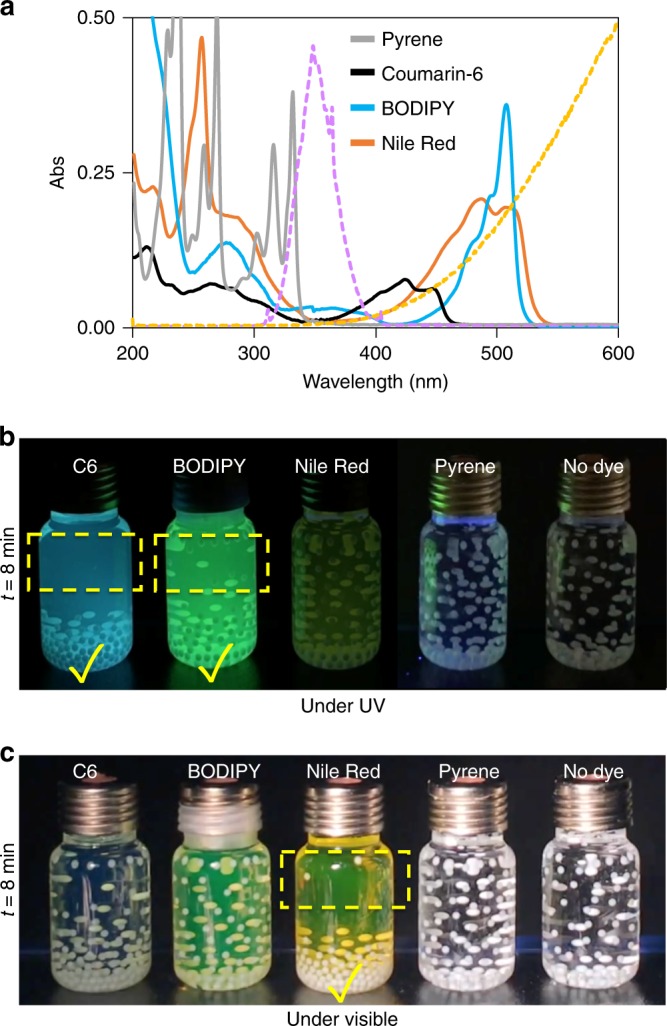
Wavelength control in discharging of tribocharges on polymers by light. **a** The emission spectra of the dyes; pyrene, Coumarin 6 (C6), (4,4-Difluoro-1,3,5,7-Tetramethyl-4-Bora-3a,4a-Diaza-s-Indacene) BODIPY, and Nile Red (all in hexane) used as mediators and the emission profiles of the light sources used in discharging ultraviolet (UV) lamp, purple dashed line (centered at ca. 350 nm) and tungsten lamp, yellow dashed line). For comparison, dye concentrations were adjusted as 1 × 10^−5^ M, 1 × 10^−5^ M, 5 × 10^−4^ M, and 1 × 10^−5^ M, respectively. **b** Forty Poly(tetrafluoroethylene) (PTFE) beads tribocharged in the dye solutions (all 1 × 10^−5^ M, hexane) in glass vials illuminated on the UV lamp with the emission profile shown in **a**. See also Supplementary Movie 3. **c** Same vials are illuminated only with visible light (tungsten lamp, profile shown in **a**). See also Supplementary Movie 4. A wavelength match between the absorption band of the dye and the emission profile of the light source is necessary for a successful discharge—however, it is not sufficient for some dyes, e.g., pyrene, see Supplementary Movie 6 and Fig. 6 for details

So far, we have shown that the photoexcitation of dyes is essential for the light-induced discharging. Therefore, the overlap between the wavelength of the light source and the absorption band of the dyes (Fig. 4a) is anticipated for fast discharging. We compared the discharge behavior of PTFE beads in pyrene, Coumarin 6 (C6), 4,4-Difluoro-1,3,5,7-Tetramethyl-4-Bora-3a,4a-Diaza-s-Indacene (BODIPY), and 9-diethylamino-5-benzo[a]phenoxazinone (Nile Red) solutions illuminated by a UV light source (Fig. 4b and Supplementary Movie 3), or by a visible light source (Fig. 4c and Supplementary Movie 4). We found out “matching wavelength” is essential for a successful discharge. However, it is quite surprising that some dyes, e.g., pyrene, cannot mediate the light induced discharging of the polymers, even when the “wavelength overlap” is provided. To understand this controversial result, we tried to clarify the mechanism of the light induced discharging as described below in the Discussion section.

### Spatial control in light induced discharging

**Fig. 5:**
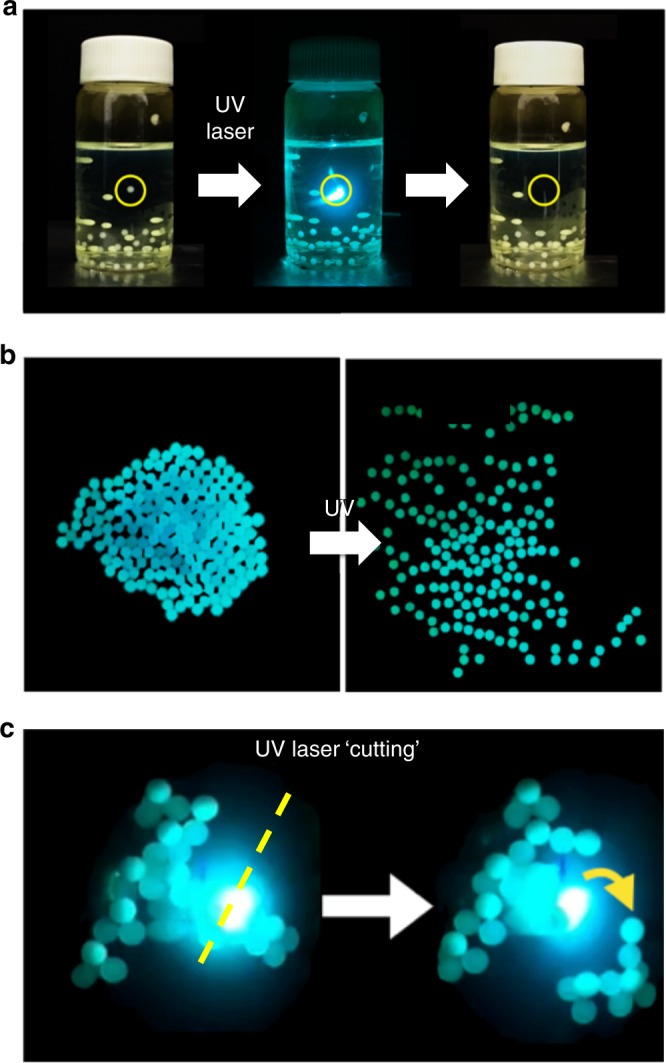
Spatial control of discharging of tribocharges on polymers by (focused) light. **a** Polymer beads charged in the dye solutions (shown here, 40 poly(tetrafluoroethylene) (PTFE) beads in 1 × 10^−5^ M Coumarin 6 (C6) in dry hexane) can be individually discharged by targeting them with a handheld laser (404 nm). **b** A 1:1 electrostatic self-assembly of PTFE and poly(oxymethylene) (POM) beads^29^ in C6 solution (1 × 10^−5^ M, dry hexane) in a gently agitated petri dish can be disassembled after discharging of the beads upon UV illumination. **c** 2D and 3D assemblies of polymer beads can be “cut” by a laser at desired locations. (Here shown, 1:1 electrostatic self-assembly of PTFE and POM beads in C6 solution (1 × 10^−5^ M, dry hexane), see also Supplementary Movie 5)

The light induced discharging process can also be spatially controlled by using well-focused light beams, e.g., lasers. We show a simple demonstration of this control; the laser pointers of 404 nm “blue”, 532 nm “green”, and 635 nm “red” wavelength and 1 mm beam size at the target were pointed on the charged PTFE beads in C6 and BODIPY solutions. As expected, only the “matching wavelength”, “blue”, and “green” pointers, affected the discharging in these solutions, and caused the targeted bead to discharge (and fall) without affecting the others (Fig. 5a). This way, all beads could be consecutively discharged and individually manipulated. In another experiment, we also showed that the laser manipulation could be extended on electrostatic self-assemblies of macro objects^29^: We first prepared an electrostatic assembly of tribocharged PTFE and POM beads in C6 solutions in glass petri dishes by manually shaking a 1:1 mixture of PTFE and POM beads for ca. 1 min, using a procedure similar to the one reported in a previous study^29^. Upon illumination by the UV light source, the beads quickly discharged and the electrostatic assembly disassembled (Fig. 5b). One might also use laser to selectively discharge beads at the desired loci of the electrostatic self-assembly, to “cut” the assembly into pieces (Fig. 5c and Supplementary Movie 5). To the best of our knowledge these experiments are the first examples of a light controlled manipulation of an electrostatically self-assembled system.

## Discussion

**Fig. 6:**
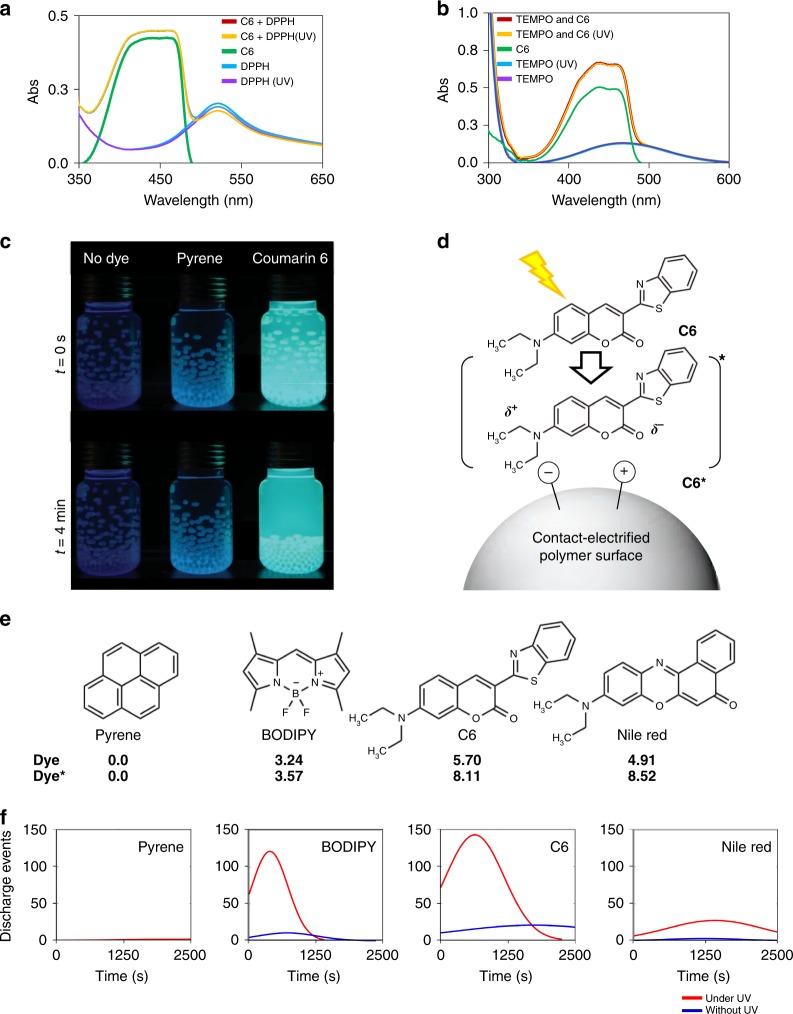
Possible interactions upon light induced discharge. **a**, **b** Dye/radical interaction or reaction: UV–Vis spectra of the 10 min ultraviolet (UV) illuminated solutions of the example radicals with (1,1-diphenyl picrylhydrazyl (DPPH) (1 × 10^−5^ M) + Coumarin 6 (C6) (1 × 10^−5^ M) and 2,2,6,6-tetramethylpiperidine 1-oxyl (TEMPO) (1 × 10^−5^ M) + C6 (1 × 10^−5^ M)) show no change with those of the mixtures before illumination. (In **a** DPPH + C6 “bleaching” is to the same extent with the bleaching of the illuminated DPPH alone). Therefore, one can say that the discharging does not involve interactions/reactions of the dyes with the radicalic species on polymers. **c** Forty poly(tetrafluoroethylene) (PTFE) beads in C6 solution (abs max 427 nm) discharge quickly when they are illuminated with UV light, whereas the same beads in pyrene solution (abs max 340 nm) do not discharge even after prolonged illumination with the same source. See also Supplementary Movie 6. (Both dyes = 1 × 10^−5^ M, dry hexane, tribocharged for 2 min). **d** An interaction between a highly polar excited state of C6 and the charges on the polymer surface may facilitate a possible electron transfer. **e** Calculated dipole moments of the photoexcited dyes in their ground and excited states. **f** Statistics from the discharging experiments shown in **c**. Data from five independent experiments was collected (a total of 200 beads for each experiment, from which only the beads displaying “discharge events” were counted); curve fit on data collected in experiments with (red) and without illumination (blue) was made by Matlab 2017 program using distribution function. For experimental details see Methods

Following the results above we attempted to find hints about the underlying mechanism of the light controlled discharging of common polymers, which would also be helpful in understanding the charging and discharging of polymers debated for decades. First of all, we should emphasize that the mechanism of light induced discharging does not involve a light-induced increase in conductivity, as proven by the surface resistivity measurements of the PDMS^12^ pieces doped with the C6 dye (Supplementary Figure 8a), or dye solutions in hexane (Supplementary Figure 8b), with and without illumination. There is also no detectable dye deposition on the beads as verified by XPS measurements (Supplementary Figure 9). Therefore, to gather insight about the mechanism of discharging and to uncover the role of dyes in the discharging process, next we tried to reveal the identity of the species, radicals or ions (charges), they are interacting with. Previously, it was shown that the mechanospecies could indeed take place in reactions such as electron transfer^17^ to other species. We^12^ and others^13^ have also found that the removal of radicals produced upon mechanical stimulus is responsible for the instability and decay of the co-produced charged species (ions). For this reason, we first suspected an interaction between the radicals on polymer surfaces and the organic dye in the excited state, upon light induced discharging in solution. Since it is not possible to directly probe the interaction of the (photoexcited) dyes and the radicals on polymer surfaces, to investigate the possible interaction between radicalic species and the dye, we conducted the following experiment: We illuminated solutions of stable radicals 1,1-diphenyl picrylhydrazyl (DPPH)^30^ and 2,2,6,6-tetramethylpiperidine 1-oxyl (TEMPO) mixed with C6 in hexane by UV light for several minutes to investigate a possible reaction or electron transfer between the radicals and the dye in the excited state. There was no change in ultraviolet–visible (UV–Vis) spectra of these mixtures upon illumination (Fig. 6a, b), and thus no reaction or electron transfer facilitated by the photoexcited dye. This implies that the C6 dye in the excited state does not react with or scavenge radicals generated on polymer surfaces. Therefore, the discharging of the polymer surfaces is not due to removal of radicals.

Eliminating the radicals from the mechanism, we turned our attention to the possible interactions between charged species (immobile polymer ions formed upon mechanical bond-breaking)^12,26^ on polymer surfaces and photoexcited dyes. At this point, we again used pyrene, BODIPY, and Nile Red to probe any differences that might be emerging from the nature of the dye. In this case, for example, photoexcited pyrene does not cause any discharging of the tribocharges on the polymer beads (Fig. 6c, f, Supplementary Movie 6) even for days, although the excitation band of the dye fits the emission spectrum of the light source (Fig. 4a). We surmise that the photoinduced discharging is more pronounced for the polymer beads in C6, BODIPY, and Nile red solutions than the ones in pyrene solution because of the differences between the polarity of the dyes; i.e., ground and excited states of pyrene are less polar (0 D) and less charge-separated than those of BODIPY, C6, and Nile red, of which the ground and excited state dipole moments were calculated to be in the range between 3.24 D–8.52 D (Fig. 6e, Supplementary Table 1). We envisage that polar dyes interact with the tribocharges on the polymer surface (as sketched in Fig. 6d) and facilitate a photo-initiated electron/energy transfer to/from the tribocharges, especially when they are in their more polar (and more reactive) excited states. The idea that (a change in) the polarity of the added dye might play a role in the discharging mechanism is supported by the facilitated discharging by other organic “impurities” of similar dipole moments, when they are present in the solution at the same concentrations as the dyes. (Supplementary Figures 10 and 11).

In this study, we show a light controlled discharge of tribocharges on common polymers through the interaction between charge-separated photoexcited dyes and the tribocharges. This new approach provides a spatial, temporal, and wavelength control for discharging of polymers. It also provides a way to manipulate small polymeric objects and their assemblies by light. We believe that our results provide new insights into a centuries-old fundamental scientific question of how tribocharges are created and can be dissipated, and also help to solve industrial problems related to electrostatic discharge.

## Methods

### Materials

All solvents were purchased from Sigma Aldrich (chromatography grade), and dried over 4 Å molecular sieves for 24 h. 3-(2-Benzothiazolyl)-7-(diethylamino)coumarin (Coumarin 6, **C6**), 9-diethylamino-5-benzo[a]phenoxazinone (Nile Red), 1,1-diphenyl picrylhydrazyl (DPPH)^30^ and 2,2,6,6- tetramethylpiperidine 1-oxyl (TEMPO) were purchased from Sigma Aldrich. The hexane-soluble 4,4-difluoro-1,3,5,7-tetramethyl-4-bora-3a,4a-diaza-s-indacene (BODIPY) dye was donated by Prof. Engin U Akkaya.

1.6 mm polytetrafluoroethylene (PTFE), poly(hexamethylene adipamide) (Nylon), polyoxymethylene (POM), or polypropylene (PP) spheres were purchased from Engineering Laboratories Inc. Prior to experiments, both polymer beads and 20 mL glass vials with PP caps were carefully washed with ethanol and dried at 50 °C overnight. To avoid contamination with dust etc., all subsequent manipulations/procedures were performed in a closed chamber. The initial charges on the beads (<10 pC) were measured using a homemade Faraday cup connected to a Keithley 6517B electrometer. The beads were placed into ordinary glass or scintillation vials, which contained one of the solvents (hexane, toluene, tetrahydrofuran (THF), chloroform (CHCl_3_)), or the solution of the dyes or other substances in dry hexane as described in the text and below.

Poly(dimethylsiloxane) (PDMS) pieces were prepared by mixing a degassed elastomer base and a crosslinker in a 10:1 w/w ratio (Sylgard 184, Dow Corning). Prepolymer mixture was cast on polystyrene petri dishes, and cured at 65 °C for 24 h. After curing the prepolymer, the PDMS pieces (ca. 1 cm × 1 cm × 0.5 cm) were gently cut and peeled off the dishes, washed with dichloromethane for 24 h (to remove catalyst and unreacted monomers) and thoroughly dried prior to the charging experiments. Doping PDMS with dyes: PDMS pieces prepared as described above were immersed into 1 × 10^−5^ M solution of Coumarin 6 in dry dichloromethane. (Higher doping concentrations may result in precipitation/recrystallization of the dye in PDMS, therefore we use the concentrations that did not yield such precipitation.) The pieces were let to swell in the dye solution for 18–24 h. The swollen polymer pieces were first dried in air and then under high vacuum for 48–96 h prior to experiments. Antistatic tweezers (Vetus, ESD-17) were used in manipulation of the pieces. Any excess charge on the pieces was removed by Zerostat antistatic instrument (Sigma-Aldrich) prior to the experiments.

### Instrumentation

For shaking, Dragon Lab MS-X vortex mixer was used. The absorption spectra were recorded using a Cary 300 UV–Visible spectrophotometer from Agilent. Discharging by UV was performed by illumination on a CAMAG laboratory UV Lamp 366 and 254 nm. Discharging by visible light was accomplished using a OSRAM HALOPAR 30 6485FL 230 V 75 W 650 lm 2900 K (Visible lamp) placed at a distance of 30 cm to the vials (IR radiation is prevented by placing a crystallization tank filled with water between the vials and the lamp). Solid PDMS pieces were treated the same unless otherwise is stated. Emission profiles of the lamps used were recorded by an Emission Spectrometer (Ocean Optics Maya2000 Pro 200–1000 nm). XPS measurements were performed using Thermo Scientific Spectrometer with monochromatized Al K-Alpha X-ray source, spot size was set to 400 μm. Humidity and temperature were recorded using a hygrometer (Traceable 37950-11, Cole-Parmer).

### Polymer bead charging experiments and data analysis

Any excess charge that might reside on the surface of the vials was removed by ethanol washing and drying at 50 °C overnight, and the vials were grounded prior to the experiments. All experiments were conducted at 25–35% relative humidity; all runs for the comparison of the discharge times were done and recorded simultaneously, under the same humidity. Glass or glass vials with inner walls covered with POM, or PET sheets (200 μ thick) are charged with hexane only, hexane solutions (1 × 10^−5^ M, 5 × 10^−5^ M, and 1 × 10^−4^ M) of **C6**, BODIPY, Nile Red, pyrene, respectively, or with DMF, H_2_O, or acetone (1 × 10^−5^ M) in hexane. The shaking parameters used in the experiments are: 2500 rpm, 0.5–2 min shaking time, 20, 40, or 60 beads. After the shaking time is over, the beads that are stuck on the walls of the vial through electrostatic adhesion were monitored by a video camera and their individual retention times on the glass vial (discharging times) were noted. Each downward motion (slipping, hopping, or falling to the bottom of the vial) of the tribocharged beads is termed as “1 discharge event”. For analysis, data from five or six independent experiments was collected (a total of 200–240 beads for each experiments, from which only the beads displaying the above-mentioned “discharged events” were counted); curve fit on data was made by Matlab 2017 program using distribution function. An example of the collected data and the curve fit can be seen in Supplementary Figure 12.

The experiments were then repeated for different shaking times, in presence of the dye solutions or pure solvents to obtain discharging time profiles.

### Charge measurement of individual beads in hexane

A homemade Faraday cup built from two concentric brass cylinders (1 cm height, and 1.2 cm and 0.8 cm radii) placed inside of the cap of the glass vial was connected to a high precision electrometer, Keithley Instruments, model 6517B. The beads were dropped one-by-one into the Faraday cup by mechanical agitation (Supplementary Figure 4).

### Charge decay experiments with PDMS pieces

All decay experiments were performed under ambient conditions (typically, temperature ~22 °C, relative humidity ~24%). A cooled chamber was used to keep the temperature constant during long illuminations of the pieces. PDMS pieces were tribocharged on an aluminum foil, and then immersed in a homemade Faraday cup (connected to a high precision electrometer, Keithley Instruments, model 6517B) to measure net charges on the contact-electrified PDMS and monitor their charge decay. Light Tech model GPH212T5L/4 8 V lamp was used to illuminate PDMS pieces in the decay experiments involving light.

### Surface resistivity measurements

Surface resistivities of PDMS, and dye-doped PDMS were measured using a two-probe method, with *w* = 5 mm wide samples, and the distance between electrodes *d* = 0.5 mm. I–V curves were collected on a Keithley electrometer (6517B), which served as the voltage source and also measured the generated current. Applied voltage was changed from 0 to 100 V in steps of 10 V. Using the slopes of the I–V curves, the values were calculated for surface resistivity, Rs, according to equation Rs = (V/I)∙(*w*/*d*). For PDMS, and dye-doped PDMS (1 × 10^−5^ M, see above for preparation details) the measured surface resistivities were determined to be ca. 10^14^ ohm sq^−1^. (Supplementary Figure 8).

### Preparation of electrostatic self-assemblies

20 PTFE and 20 POM beads were introduced into a 1 × 10^−5^ M, dry hexane Coumarin 6 solution in a glass petri dish, and the mixture was shaken for 1 min, until which time the assembly was formed. The assembly was then illuminated with UV light to discharge and disassemble (Fig. 5).

### Electronic structure calculations

Ground and excited state calculations were performed at UB3LYP/6-31 + G(d) level of theory. Vibrational frequency calculations ensured that located structures correspond to true minima, i.e., eigenvalues of the Hessian matrix were positive. Gaussian 09 software suite was used throughout the calculations. Geometry optimizations and time dependent density functional theory (TD-DFT) excitations were calculated with UB3LYP/6-31 + G(d) level of theory.

## Supplementary information

Supplementary Information

Description of Additional Supplementary Files

Supplementary Movie 1

Supplementary Movie 2

Supplementary Movie 3

Supplementary Movie 4

Supplementary Movie 5

Supplementary Movie 6

Source Data

## Data Availability

The datasets generated during and/or analysed during the current study are available from the corresponding author on reasonable request. The source data underlying Figs. 1b, 2d, and 3a are provided as a Source Data file.
