# Supplementary material for: Control of triboelectric charges on common polymers by photoexcitation of organic dyes
Source: Nat Commun. 2019 Jan 17;10:276. doi: 10.1038/s41467-018-08037-5 (PMC6336862; doi:10.1038/s41467-018-08037-5)
Supplement: Supplementary file 1 — Supplementary Information [file 41467_2018_8037_MOESM1_ESM.pdf]

Supplementary Information

Control of Triboelectric Charges on Common Polymers by Photoexcitation of Organic Dyes

Cezan et al.

Supplementary Figures

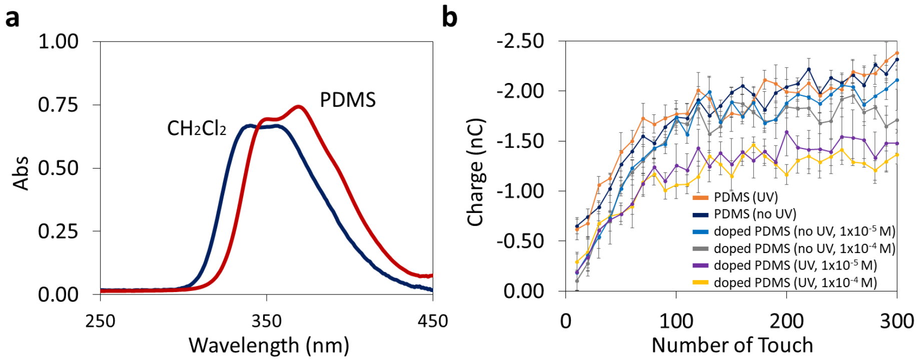

Supplementary Figure 1. Ultraviolet-visible (UV-Vis) spectrum of the Coumarin 6 (**C6)** dye in C6-doped PDMS and tribocharging of the C6-doped poly(dimethylsiloxane) PDMS pieces with and without illumination. a) UV-Vis spectrum of the **C6** dye in CH_2Cl_2 ($1x10^{-5}$ M) and in PDMS (**C6** doping by letting the immersed piece in $1x10^{-5}$ M **C6** solution in CH_2Cl_2). b) **C6**-doped (1×10^{-5} M, 1×10^{-4} M) and undoped PDMS pieces (1 cm x 1 cm surface area) are contact-charged by touching to an aluminium foil and tribocharges on the pieces are recorded by using a homemade Faraday cup connected to an electrometer, immediately after each touch. Overall, the doped pieces acquire slightly less charges than the non-doped pieces presumably due to a 'medium effect' that is induced by the doping of the polymer with the polar dye. The undoped PDMS pieces illuminated with UV light show similar charging with or without illumination, however, the doped ones show a decrease in the net charge upon illumination-accompanied charging. Error bars correspond to standard deviations determined from at least four independent experiments. See Methods for further experimental details on sample preparation and Supplementary Fig. 2 for data acquired from charge decay experiments.

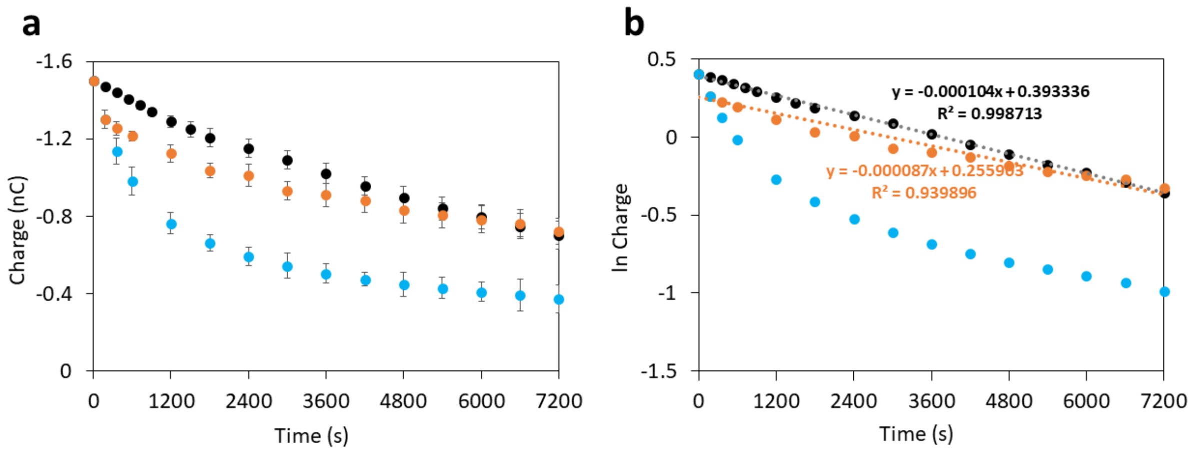

Supplementary Figure 2. a) The magnitudes of charges on the Coumarin (**C6)** (1 x 10^{-5} M) doped poly(dimethylsiloxane) PDMS under ultraviolet (UV) light (blue), decay faster than those on PDMS under UV and ambient light (black, the two lines of data overlap), and those on **C6** doped PDMS under ambient light (green). The decay rate constants are determined from the slopes of the semi-logarithmic plots as shown in (b). These rate constants are 1.04 x10^{-4} s^{-1} for PDMS under UV and ambient light, and 0.87 x10^{-4} s^{-1} for **C6** doped PDMS under ambient light. The decay of charges on **C6** doped PDMS under UV light (blue) deviate from a first order scheme, showing an involvement of another species (in this case, the dye) in the discharging mechanism. Error bars correspond to standard deviations determined from at least four independent experiments for every condition. Decay rate calculations were done on OriginPro09 by fitting first order decay curve. See Methods for details of sample preparation.

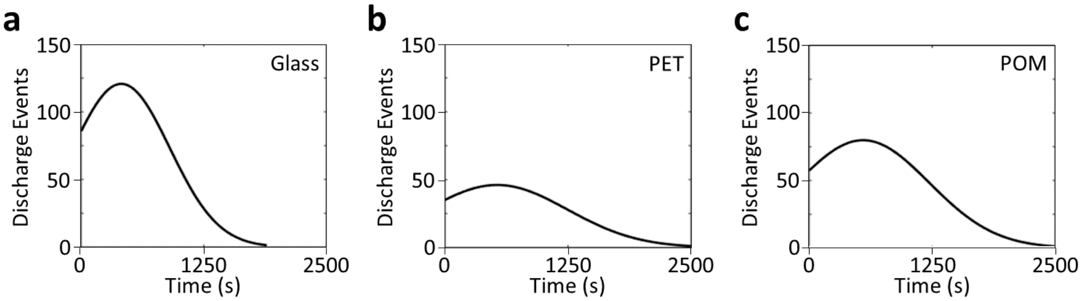

Supplementary Figure 3. Tribocharged polymer beads (here data shown for poly(tetrafluoroethylene) (PTFE) beads) discharge faster in UV-illuminated Coumarin 6 (**C6**) solution in a) glass vials, and glass vials with b) poly(oxymethylene) POM, and c) poly(ethyleneterephthalate) PET covered inner walls than in hexane only (Fig 2c, main text) in all of these vials). [**C6**]=1 x 10^{-5} M. Data from five independent experiments was collected (a total of 200 beads for each experiment, from which only the beads displaying 'discharge events' were counted); curve fit on data collected in experiments was made by Matlab 2017 program using distribution function. For experimental details see Methods.

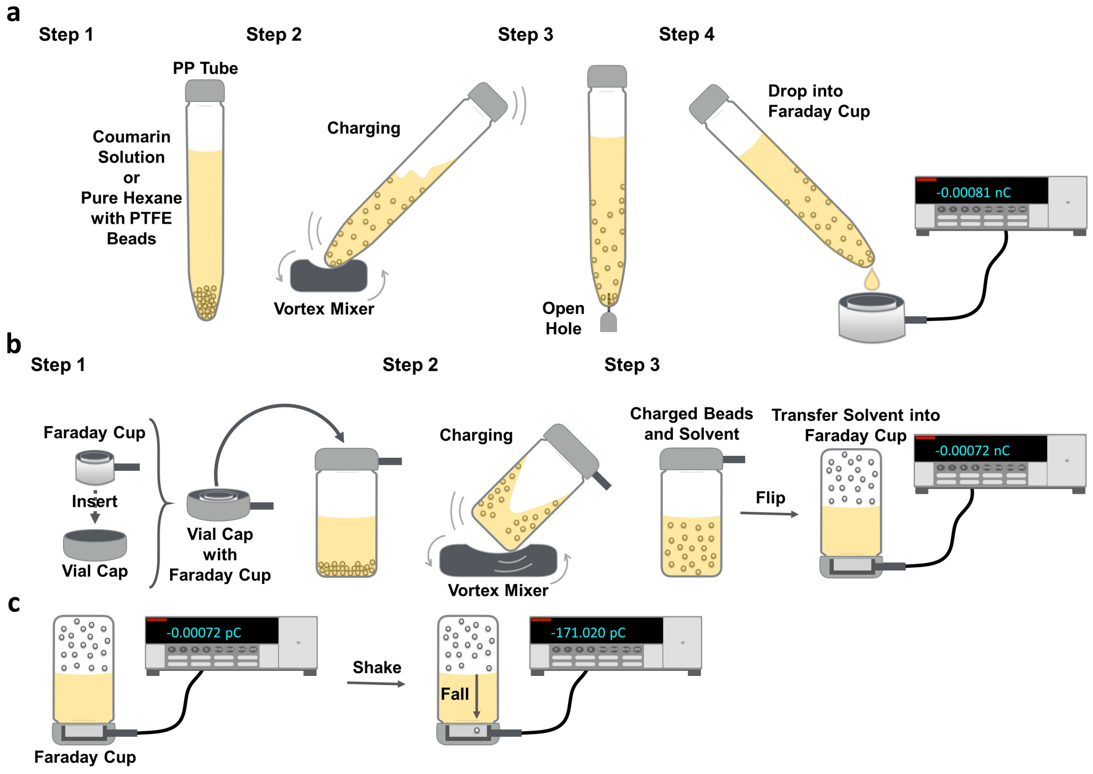

Supplementary Figure 4. Experimental setup for determination of possible tribocharging in hexane during vortexing of the polymer beads in hexane and in Coumarin (**C6**) solutions and measurement of charges of individual beads. a) 40 poly(tetrafluoroethylene) PTFE beads in hexane or in a **C6** solution (5 x 10^{-4} M, hexane) are tribocharged by shaking on a vortexer for 1 min. b) The charged beads 'stick' electrostatically to the walls of the polypropylene (PP) centrifuge tube and stay stuck on the walls for hours to days. Through a small hole cut at the bottom of the PP tube, hexane or **C6** solution was dropped into a Faraday cup connected to an electrometer. b) A homemade Faraday cup was attached inside of the vial cap of a glass vial containing 40 PTFE beads and hexane or **C6** solution (5 x 10^{-4} M, hexane). After tribocharging by vortexing, the vial was turned upside down to let the solvent or solution into the Faraday cup. In both (a) and (b) the overall net charge of hexane was found to be <20 pC, whereas in c) when the tribocharged beads were let to fall one-by-one into the Faraday cup in the vial cap, the charge on beads were measured to be -170 ± 55 pC.

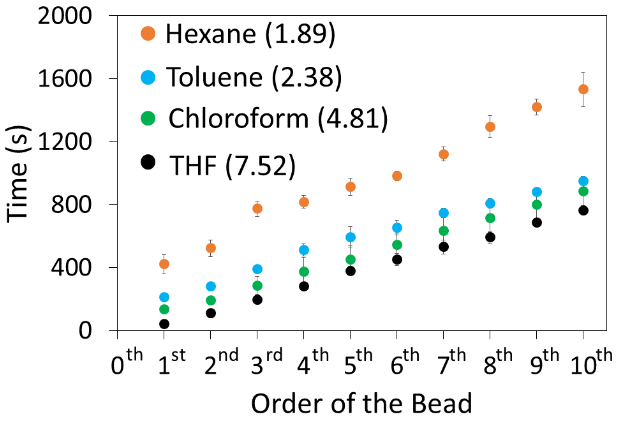

Supplementary Figure 5. Discharging times of the tribocharged polymer beads in solvent-filled glass vials is dependent on the dielectric constant of the solvent. Discharging times of the individual beads (40 poly(tetrafluoroethylene) PTFE beads in dye solution, shaken 1 min in vortexer) increase with decreasing dielectric constants (in parenthesis) of the solvents; ζhexane (1.89)> ζtoluene (2.38) > ζchloroform (4.81) > ζTHF (7.52). Error bars on the discharging times correspond to standard deviations determined from at least six independent experiments.

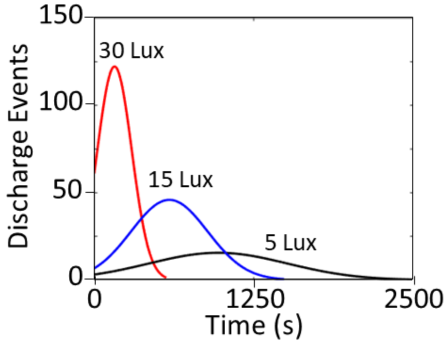

Supplementary Figure 6. Tribocharged polymer beads (here data shown for poly(tetrafluoroethylene) (PTFE)) discharge faster in UV-illuminated Coumarin 6 (**C6)** (1 x 10^{-5} M in hexane) solution in glass vials when the intensity of the light is increased from 5 Lux to15 Lux, and then to 30 Lux. Data from five independent experiments was collected (a total of 200 beads for each experiment, from which only the beads displaying ‘discharge events’ were counted); curve fit on data collected in experiments was made by Matlab 2017 program using distribution function. See also Supplementary Figure 12 for the details of curve fitting on the data. See Methods and main text for further experimental details about the charging/discharging experiments and curve fitting.

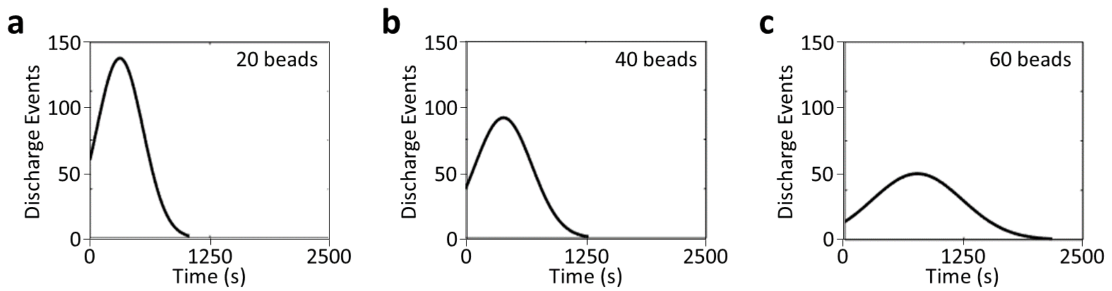

Supplementary Figure 7. Effect of the number of polymer beads on discharging time profiles of the tribocharged polymer beads. a) 20, b) 40, c) 60 tribocharged polymer beads (here data shown for poly(tetrafluoroethylene) (PTFE)) discharge faster in UV-illuminated Coumarin 6 (**C6)** solution in a) glass vials than in hexane only (Fig 2c, main text) in all of these vials. [**C6**]=1 x 10^{-5} M. Data from five independent experiments (a total of 200 beads for each experiment, from which only the beads displaying the above-mentioned 'discharged events' were counted) was collected; curve fit on data collected in experiments with (red) and without illumination (blue) was made by Matlab 2017 program using distribution function. For experimental details see Methods section in the main text.

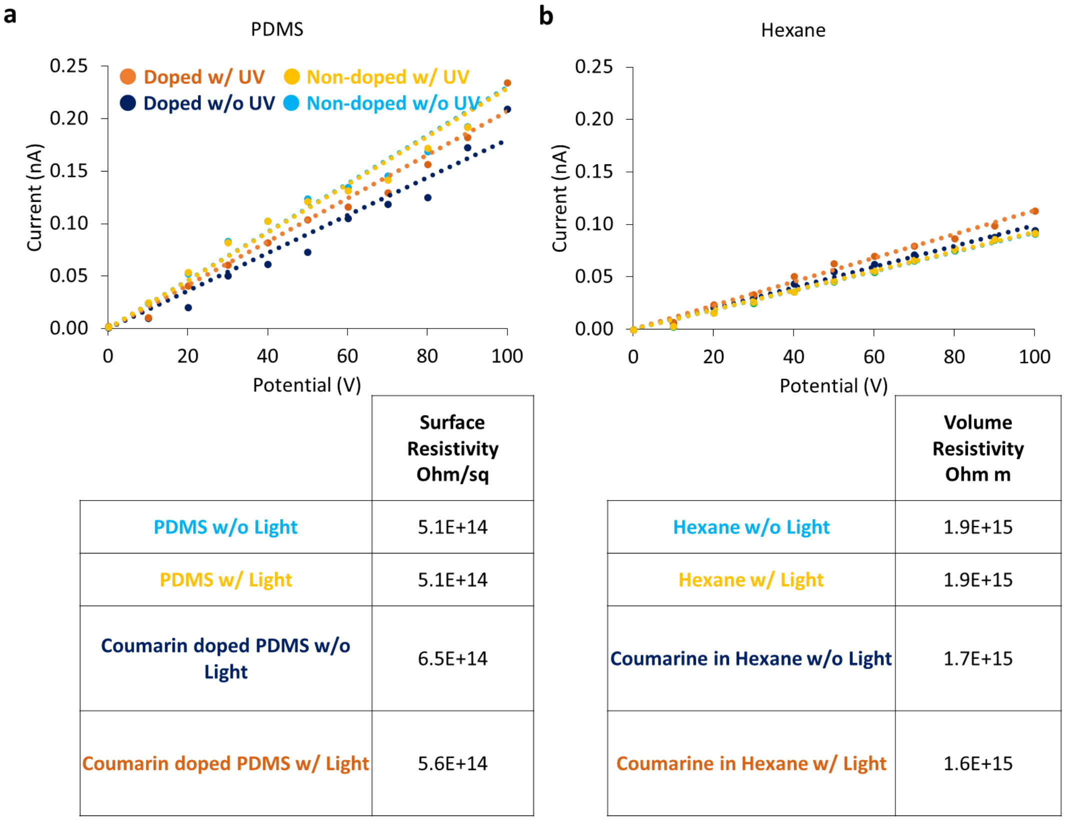

	Surface Resistivity Ohm/sq
PDMS w/o Light	5.1E+14
PDMS w/ Light	5.1E+14
Coumarin doped PDMS w/o Light	6.5E+14
Coumarin doped PDMS w/ Light	5.6E+14

	Volume Resistivity Ohm m
Hexane w/o Light	1.9E+15
Hexane w/ Light	1.9E+15
Coumarine in Hexane w/o Light	1.7E+15
Coumarine in Hexane w/ Light	1.6E+15

Supplementary Figure 8. a) I-V curves and the calculated surface resistivities of poly(dimethylsiloxane) PDMS and dye doped PDMS pieces (1 x 10^{-5} M) (See also Fig 1 in the main text and Methods). b) I-V curves and the calculated volume resistivities of hexane and coumarin in hexane (5x10^{-4} M) (See Fig. 2 in the main text and methods) with and without illumination with ultraviolet (UV) light.

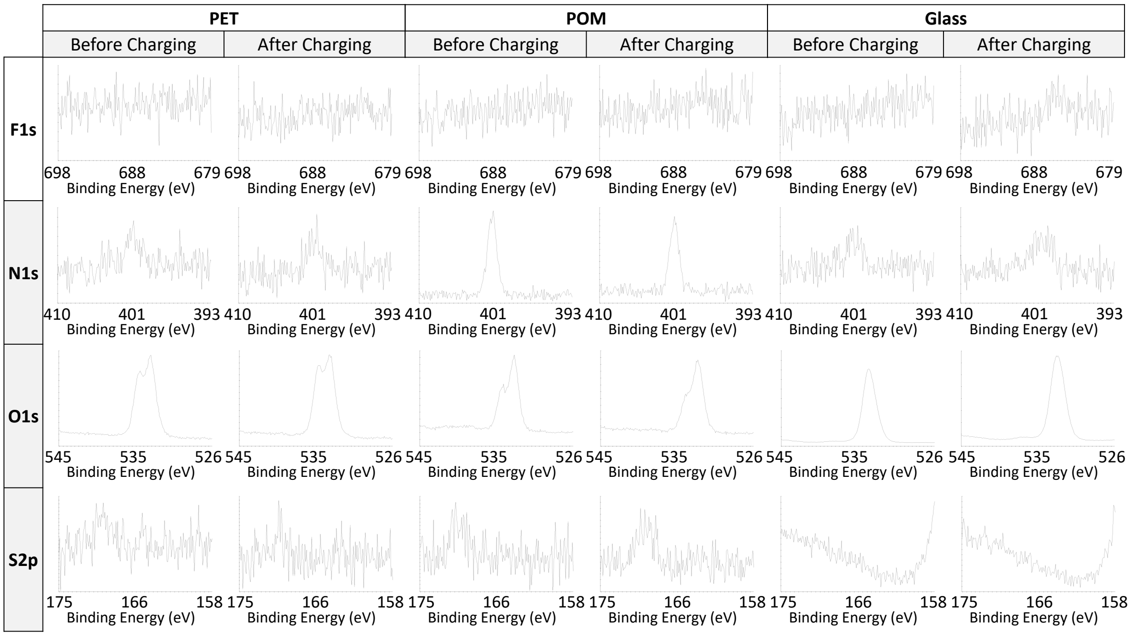

Supplementary Figure 9. XPS analysis of the polymer bead surfaces before and after tribocharging. 40 poly(tetrafluoroethylene) (PTFE) beads in coumarin 6 (**C6**) solutions (5 x 10^{-4} M, dry hexane) (right) in glass vials (native glass vials, and glass vials with poly(oxymethleyene) (POM)-, and poly(ethyleneterephthalate) (PET)-covered inner walls) were tribocharged by shaking on a vortexer for 1 min and discharged by a UV lamp. There is no significant dye adsorption on the beads as seen from the N1s, O1s, and S2p signals acquired from the polymer beads and on the glass vial before and after tribocharging. See *Methods* for experimental details on XPS measurements.

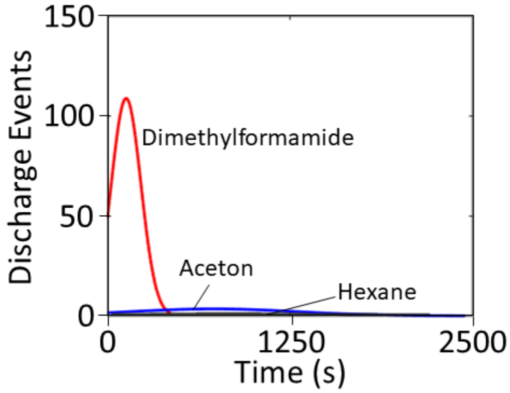

Supplementary Figure 10. Tribocharged poly(tetrafluoroethylene) (PTFE) beads discharge faster in hexane solutions with 5 x10^{-5} M DMF (added as a polar impurity) with dipole moment of 3.86 D (data in red curve) than 5 x10^{-5} M acetone with dipole moment of 2.88 D (data in blue curve). Data in pure hexane is shown with the black curve, with almost no discharging at the monitored time interval. Data from five independent experiments was collected (a total of 200 beads for each experiment, from which only the beads displaying 'discharge events' were counted); curve fit on data collected in experiments was made by Matlab 2017 program using distribution function. For experimental details see *Methods*.

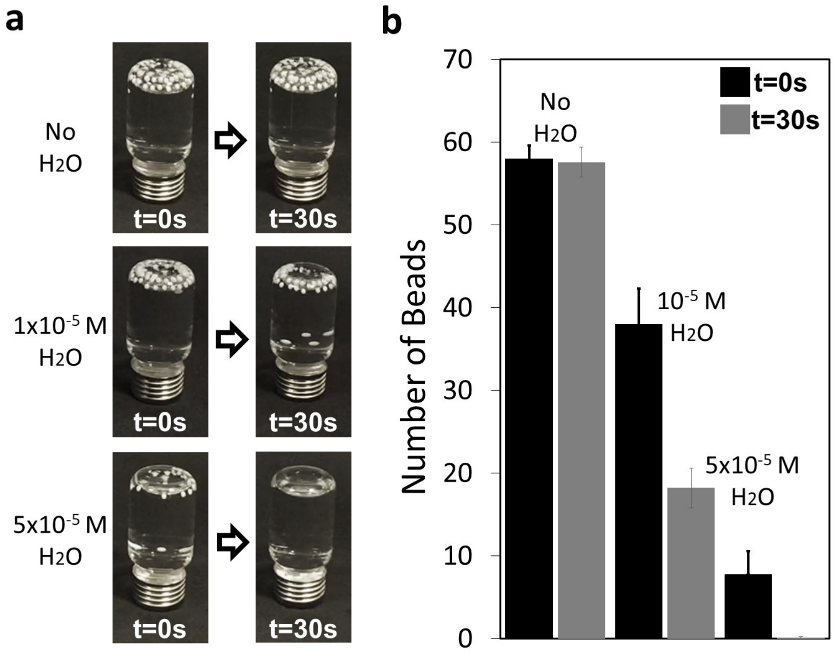

Supplementary Figure 11. Experimental setup for determination of role of water in tribocharging of polymer beads in hexane. a) 60 poly(tetrafluoroethylene) (PTFE) beads in hexane are tribocharged by shaking on a vortexer for 1 min. The charged beads ‘stick’ electrostatically to the walls of the glass vial (especially to the bottom of the vial as shown in the upside down view of the vials in the photo) stay stuck on the walls for hours. b) The number of beads that stay charged at t=0 s and t=30 s after tribocharging at different concentrations of added water.

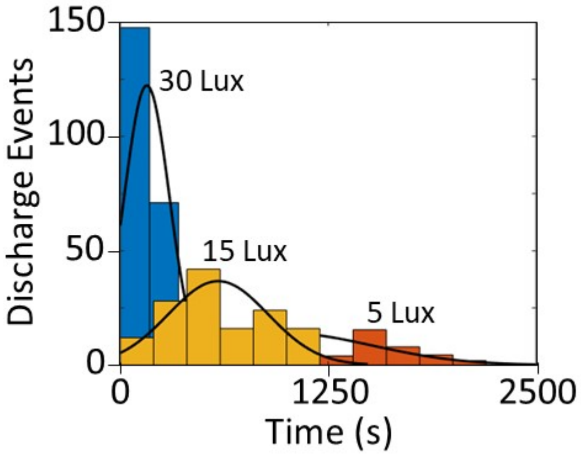

Supplementary Figure 12. Tribocharged polymer beads (here data shown for poly(tetrafluoroethylene) (PTFE)) discharge faster in UV-illuminated Coumarin 6 (C6) solution in glass vials when the intensity of the light is increased from 5 Lux to15 Lux and then to 30 Lux (data plotted as the black curves only, in Supplementary Fig 5). Data from five independent experiments (a total of 200 beads for each experiment, from which only the beads displaying the above-mentioned 'discharged events' were counted) was collected and plotted as bar graph in the given time intervals; curve fit on the bar data was made by Matlab 2017 program using distribution function. For experimental details see *Methods*.

	CAM-B3LYP/6-31G		
Dye	S_0 (DFT)	S_1 (TD-DFT)	Δ S_1-S_0
Pyrene	0	0	0.00
BODIPY	3.24	3.88	0.64
Coumarin 6	5.70	8.15	2.45
Nile Red	4.91	8.52	3.61

Supplementary Table 1. Calculated Dipole Moments (Debye) and Differences for Ground (S_0) and Excited State (S_1)**.** Ground and excited state calculations were performed at CAM-B3LYP/6-31G level of theory.
