# Supplementary material for: Control of triboelectric charges on common polymers by photoexcitation of organic dyes
Source: Nat Commun. 2019 Jan 17;10:276. doi: 10.1038/s41467-018-08037-5 (PMC6336862; doi:10.1038/s41467-018-08037-5)
Supplement: Supplementary file 2 — Description of Additional Supplementary Files [file 41467_2018_8037_MOESM2_ESM.pdf]

Description of Additional Supplementary Files

File Name: Supplementary Movie 1
Description: Typical vortexing of poly(tetrafluoroethylene) (PTFE) beads for tribocharging. Here, 40 PTFE beads are tribocharged in a Coumarin 6 (C6) solution (5 x 10-4 M, hexane) by shaking using a vortexer.

File Name: Supplementary Movie 2
Description: Light controlled discharging of tribocharged polymer beads. 40 poly(tetrafluoroethylene) PTFE beads in hexane (left) and in a Coumarin 6 (C6) solution (5 x 10-4 M, hexane) (right) are tribocharged by shaking on a vortexer for 1 min and placed on a ultraviolet (UV) lamp. The charged beads 'stick' electrostatically to the walls of the vials and stay stuck on the walls for hours to days. When the UV lamp is turned on, only the beads in C6 solution discharge (within a few minutes) and fall to the bottom of the vial. For experimental details see Methods.

File Name: Supplementary Movie 3
Description: Wavelength control in light controlled discharging of tribocharged polymer beads. 40 poly(tetrafluoroethylene) (PTFE) beads tribocharged in the pyrene, Coumarin 6 (C6), 4,4-Difluoro-1,3,5,7-Tetramethyl-4-Bora-3a,4a-Diaza-sIndacene (BODIPY), and 9-diethylamino-5-benzo[a]phenoxazinone (Nile Red) dye solutions (all 1x10-5 M, hexane) in glass vials illuminated on the UV lamp. A wavelength match between the absorption band of the dye and the emission profile of the light source is necessary for a successful discharge (see also Fig 4a) – however, it is not sufficient for some dyes, e.g. pyrene, see Supplementary Movie 4 and Fig 6 for detailed discussion.

File Name: Supplementary Movie 4
Description: Wavelength control in light controlled discharging of tribocharged polymer beads. 40 poly(tetrafluoroethylene) (PTFE) beads tribocharged in the pyrene, , Coumarin 6 (C6), 4,4-Difluoro-1,3,5,7-Tetramethyl-4-Bora-3a,4a-Diaza-sIndacene (BODIPY), and 9-diethylamino-5-benzo[a]phenoxazinone (Nile Red) dye solutions (all 1x10-5 M, hexane) in glass vials illuminated with a visible light source. 3 Charge on the tribocharged polymer beads decay faster in solutions of the dyes with a noticeable visible light absorption.

File Name: Supplementary Movie 5
Description: Spatial control of discharging of tribocharges on polymers by (focused) light. Polymer beads charged in the dye solutions (shown here, 40 poly(tetrafluoroethylene) (PTFE) beads in 1x10-5 M , Coumarin 6 (C6) in dry hexane) can be individually discharged by targeting them with a handheld laser (404 nm). A 3- dimensional 1:1 electrostatic self-assembly of PTFE and poly(oxymethylene) (POM) beads in C6 solution (1x10-5 M, dry hexane) can be 'cut' by a laser at desired locations. (Here shown, 1:1 electrostatic self-assembly of PTFE and POM beads in C6 solution (1x10-5 M, dry hexane).

File Name: Supplementary Movie 6
Description: Tribocharged poly(tetrafluoroethylene) (PTFE) beads in C6 solution (abs max 427 nm) discharge quickly when they are illuminated with UV light, whereas the same beads in pyrene solution (abs max 340 nm) do not discharge even after prolonged illumination with the same source. (Both dyes=1x10-5 M, dry hexane, beads tribocharged for 2 min).
